# Tabriz nephro-educational courses; A global scientific vision with local vigilance

**DOI:** 10.12861/jrip.2014.04

**Published:** 2014-12-01

**Authors:** Mohammad-Reza Ardalan

**Affiliations:** ^1^Chronic Kidney Disease Research Center, Tabriz University of Medical Sciences, Tabriz, Iran

**Keywords:** Nephropathology, Chronic kidney disease, Nephrotoxins

**Implication for health policy/practice/research/medical education:**

New generation of physician is not aware about the epidemiology of common disease in their region. It is very important needs to combine the global scientific vision with local vigilance for health personals. Collaboration between regional centers is very important to expand the educational, preventive and research programs. These regional collaborations not only expand the sciences of nephrology in the region but also help to have scientific achievements with global impacts.

Chronic Kidney Disease Research Center (CKDRC) of Tabriz University of Medical Sciences has started its first international educational course namely: symposium and workshop of nepheopathology on in 15-17 October 2013. During these three days of teaching course, a team of Iranian pathologist in collaboration with Professor Franco Ferarrio (a reputed international nephropathologist) covered the main topics in the field of nephropathology. The main discussed area were glomerular diseases, ANCA-associated vacuities, diabetic nephropathy, lupus nephritis, renal involvement in para-proteinemia and IgA-nephropathy. The course was addressed to regional and national nephrologists and pathologists and our guests were came from different centers. During these three days of meeting, we had a vibrant environment for clinico-pathological discussions. A series of lectures, panel discussion and interactive clinic-pathological sessions were taken place, and during the interactive sessions clinical and pathologic findings were putted together. The sessions were opened to parallel question and answer and participants were able to share their opinions and ask questions of speakers and also to advice on clinical and pathologic issues.We collected remarkable positive feedbacks from participant. Lectures slides and audio-video records were collected and prepared in a special educational package and will be distributed internationally.

We believe that it is very important to make a close collaboration between nephrologist and pathologist during a special meeting which is devoted to nephropathology. During conventional pathology or nephrology conferences only one day or half a day is devoted to nephropathology, but during our recent teaching course we had three days just focusing on nephropathology. We decided to continue our teaching courses annually with international speakers and audiences in our center which is located in Azerbaijan province in north-west of Iran. Our province has common borders with Turkish, Republic of Azerbaijan, Armenia, and Georgia. These vicinities make us a potential center for regional collaboration and educational activities and to connect with international nephrology organizations. Our major education context would be nephropathology, preventive nephrology, peritoneal dialysis, hemodialysis, transplantation in developing countries, nutrition and renal disease, genetic and nephrology. We also have special focuses on diseases that are endemic in the region. Renal involvement in familial Mediterranean fever (FMF) is a common problem in our area (1-3), and sometime it presents with a confusing features (4,5). Hemorrhagic fever with renal syndromes (6-8) and Behçet’s disease are common (9-11). Infection related glomerulonephritis is an important problem (12-14), and nutritional issues need special consideration.

In our region there are nomads that have no fixed residency and move from place to place, usually seasonally, to find land for their herds. They often living in mountain region, and use high amount of salt as food preservers. We made a preliminary pilot study and found high amount of salt, and phosphate intake, high prevalence of hypertension and hypertension related complications among this population [Author’s unpublished data]. In our region, we have high prevalence of tuberculosis, sometime presenting with very unusual pictures (15). Brucellosis still is a common health problem in our region (12). In developing countries, there are wave of migration from rural areas to cities, and they are often settling in slam areas. They often change their dietary habits and using more salty fast food and also entering a sedentary lifestyle, while some of them harboring chronic infectious diseases. Very importantly, it is very difficult to expand the preventive and educational programs among these populations. Transplantation programs are expanding, and transplant induced immunosuppression creates unusual and protean face for some chronic infectious diseases that are endemic in our region (16-18). There are an extensive movement toward the natural remedies and traditional therapies in our area while some of them are potentially nephrotoxic and have profound adverse effects (19-21). Expanding the public awareness about the environmental nephrotoxins toxins has an utmost priority.

New generation of physician is not aware about the epidemiology of common disease in their region. It is very important needs to combine the global scientific vision with local vigilance for health personals. Collaboration between regional centers is very important to expand the educational, preventive and research programs. These regional collaborations not only expand the sciences of nephrology in the region but also help to have scientific achievements with global impacts.

For all above reasons, we are planning to continue our nephrology teaching course every year. Each year we are focusing on special areas with a comprehensive review. We hope that a large number of delegates, both nationally and internationally, join us every year in order to become a classic appointment for the experts. Our upcoming symposium would be held with the provisional date of October 2014. “Nephology problems in northwest of Iran and neighboring Caucasia” would be the topic of our next symposium and we try to invite international and regional authorities to discuss on the major topics in a three days of meeting. Please visit the website of Chronic Kidney Disease Research Center “www.ckdrc.tbzmed.ac.ir” for featuring updates and information about our activities.

## Author’s contribution

MRA was the single author of the manuscript.

## Conflict of interests

The author declared no competing interests.

## Ethical consideration

Ethical issues (including plagiarism, misconduct, data fabrication, informed consent, double publication) have been completely observed by the authors.

## Funding/Support

None.
